# Baseline ^18^F-FDG PET/CT Radiomics in Classical Hodgkin’s Lymphoma: The Predictive Role of the Largest and the Hottest Lesions

**DOI:** 10.3390/diagnostics13081391

**Published:** 2023-04-11

**Authors:** Elizabeth Katherine Anna Triumbari, Roberto Gatta, Elena Maiolo, Marco De Summa, Luca Boldrini, Marius E. Mayerhoefer, Stefan Hohaus, Lorenzo Nardo, David Morland, Salvatore Annunziata

**Affiliations:** 1 Section of Nuclear Medicine, Department of Radiological Sciences and Hematology, Università Cattolica del Sacro Cuore, 00168 Rome, Italy; elizakat@virgilio.it; 2 Department of Radiology, UC Davis, Sacramento, CA 95817, USA; lnardo@ucdavis.edu; 3 Department of Clinical and Experimental Sciences, University of Brescia, 25121 Brescia, Italy; roberto.gatta.bs@gmail.com; 4 Department of Oncology, Lausanne University Hospital, 1011 Lausanne, Switzerland; 5 Radiomics, Dipartimento di Radiologia, Radioterapia ed Ematologia, Fondazione Policlinico Universitario A. Gemelli, IRCCS, 00168 Roma, Italy; luca.boldrini@policlinicogemelli.it; 6 Ematologia, Dipartimento di Radiologia, Radioterapia ed Ematologia, Fondazione Policlinico Universitario A. Gemelli, IRCCS, 00168 Roma, Italy; elenam86@hotmail.it; 7 Medipass S.p.a. Integrative Service PET/CT–Radiofarmacy TracerGLab, Fondazione Policlinico Universitario A. Gemelli, IRCCS, 00168 Roma, Italy; marco.desumma@gmail.com; 8 Division of General and Pediatric Radiology, Department of Biomedical Imaging and Image-Guided Therapy, Medical University of Vienna, 1090 Wien, Austria; marius.mayerhoefer@meduniwien.ac.at; 9 Department of Radiology, Memorial Sloan Kettering Cancer Center, New York, NY 10065, USA; 10 Hematology Section, Department of Radiological Sciences and Hematology, Università Cattolica del Sacro Cuore, 00168 Roma, Italy; 11 Unità di Medicina Nucleare, GSTeP Radiofarmacia, TracerGLab, Dipartimento di Radiologia, Radioterapia ed Ematologia, Fondazione Policlinico Universitario A. Gemelli, IRCCS, 00168 Roma, Italy; salvatore.annunziata@policlinicogemelli.it; 12 Médecine Nucléaire, Institut Godinot, 51100 Reims, France; 13 CReSTIC EA 3804 et Laboratoire de Biophysique, Université de Reims Champagne-Ardenne, 51100 Reims, France

**Keywords:** classical Hodgkin’s lymphoma, ^18^F-FDG PET/CT, radiomics

## Abstract

This study investigated the predictive role of baseline ^18^F-FDG PET/CT (bPET/CT) radiomics from two distinct target lesions in patients with classical Hodgkin’s lymphoma (cHL). cHL patients examined with bPET/CT and interim PET/CT between 2010 and 2019 were retrospectively included. Two bPET/CT target lesions were selected for radiomic feature extraction: Lesion_A, with the largest axial diameter, and Lesion_B, with the highest SUV_max_. Deauville score at interim PET/CT (DS) and 24-month progression-free-survival (PFS) were recorded. Mann–Whitney test identified the most promising image features (*p* < 0.05) from both lesions with regards to DS and PFS; all possible radiomic bivariate models were then built through a logistic regression analysis and trained/tested with a cross-fold validation test. The best bivariate models were selected based on their mean area under curve (mAUC). A total of 227 cHL patients were included. The best models for DS prediction had 0.78 ± 0.05 maximum mAUC, with a predominant contribution of Lesion_A features to the combinations. The best models for 24-month PFS prediction reached 0.74 ± 0.12 mAUC and mainly depended on Lesion_B features. bFDG-PET/CT radiomic features from the largest and hottest lesions in patients with cHL may provide relevant information in terms of early response-to-treatment and prognosis, thus representing an earlier and stronger decision-making support for therapeutic strategies. External validations of the proposed model are planned.

## 1. Introduction

Hodgkin’s lymphoma (HL) is a rare B-cell malignancy with an estimated incidence of 2.3 to 2.6 cases per 100,000 people per year [1,2]. The so-called “classical” HLs (cHL) represent the vast majority of HL cases (about 95%) and are distinguished from nodular lymphocyte-predominant types due the indolent presentation and more favorable prognosis of the latter [1,3]. Nowadays, ^18^F-FDG PET/CT plays a central role in the management of this disease. Baseline ^18^F-FDG PET/CT (bPET/CT) enables patients’ risk stratification [1,4,5,6], as five-year relative survival rate relies strongly on disease stage [2] but also allows therapeutic planification [3,5]. Interim PET/CT (iPET/CT), performed after two to four courses of primary chemotherapy (PCT), has proven its usefulness, as numerous trials have used an iPET/CT response-adapted approach to evaluate early escalation or de-escalation of therapy [7]. In particular, the 5-point Deauville score (DS) derived from iPET/CT has proven high prognostic efficacy [8,9].

Despite recent therapeutical advances [10], about 25% of HL patients still relapse or die because of disease progression [1,11], calling for new prognostic factors to be found, particularly on bPET/CT. Radiomics, an emerging field of research aiming to extract mineable, quantitative, high-dimensional data from clinical images, has been suggested as a solution [12,13,14]. Various studies have explored the possibilities offered by radiomics in HL [15] whether for computer-aided histological classification [16], staging [17,18], early metabolic response assessment [19,20,21], or refractory disease prediction [22,23].

The extraction of parameters and the construction of radiomic models is a long and complex process requiring a rigorous approach. Several methodological frameworks have been proposed [16,24]. The variability of target volume delineation protocols, the lack of validation cohorts, and the lack of methodological harmonization are factors that limit the diffusion of this type of approach in clinical routine [13,25,26].

This study aimed at investigating whether bPET/CT radiomic models, which are derived from two distinct and easy-to-identify target lesions (the largest and the hottest), could predict tumor aggressiveness and patients’ prognosis considering early response to PCT (DS at iPET/CT) and progression-free survival (PFS) in a large monocentric cohort of cHL patients. In addition, inter-scanner performance differences were assessed.

## 2. Materials and Methods

### 2.1. Study Design, Patients, and Data Collection

This retrospective study was approved by the Ethical Committee of Fondazione Policlinico Universitario A. Gemelli IRCCS (study code 3834), and all included subjects signed an informed consent form.

Medical records of all patients consecutively diagnosed with HL and referred to the hematology unit between September 2010 and October 2019 were reviewed. Patients were included if they had undergone a bPET/CT and an iPET/CT after the first two cycles of PCT and had an available clinical follow-up of at least 2 years. Exclusion criteria were LPHL histology, presence of other synchronous/metachronous tumors, extensive surgical resection of disease for diagnostic purposes before bPET/CT, and first evaluation at disease relapse.

### 2.2. Image Acquisition Protocol

PET/CT studies were acquired according to European Association of Nuclear Medicine guidelines [27]. Patients fasted for ≥6 h, and their blood glucose levels were <200 mg/dL before administration of 236 ± 50 MBq of ^18^F-FDG. Images were acquired after 60 ± 10 min of uptake time using a Gemini GXL (Philips Healthcare, Cleveland, OH, USA) or a Biograph mCT (Siemens Healthineers, Erlangen, Germany) PET/CT scanner, applying the respective standard reconstruction protocol (Table 1) [27].

### 2.3. Image Segmentation and Radiomic Features’ Extraction

PET/CT images were reviewed by two experienced nuclear medicine physicians blinded to patients’ clinical and follow-up data.

**Figure 1:**
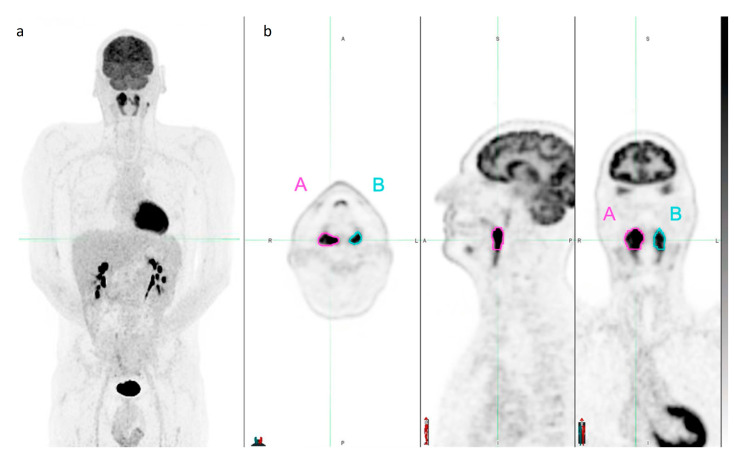
Example of target lesion contouring with PET_Edge_ tool from MIM Encore Software. (**a**) Maximum-intensity projection of a 44-year-old male patient with Stage II nodular sclerosing Hodgkin’s lymphoma. This case of rare tonsillar lymphoma was chosen to easily show contouring of two lesions on the same slice. Axial, sagittal, and coronal PET views (**b**) of Lesion_A (pink-contoured volume of interest, D_max_ 2.7 cm) and Lesion_B (cyan-contoured volume of interest, SUV_max_ 18.8).

For target lesion contouring, a semiautomatic gradient-based segmentation tool (PET_Edge_, version 7.0.5 of MIM Encore Software Inc., Cleveland, OH, USA) [28,29] was used to delineate volumes of interest (VOIs) on two distinct nodal lesions for each bPET/CT scan (Figure 1). Target Lesion_A was the lesion with the largest axial diameter (D_max_) identified on CT images. When a bulky tumor was present, Lesion_A was identified as the single distinct lesion with D_max_ among contiguous lesions in the bulk. Target Lesion_B was the lesion with the highest SUV_max_. When more than one lesion visually showed similar ^18^F-FDG uptake, a VOI was drawn around each one to choose the hottest. The conventional parameters D_max_, SUV_max_, SUV_mean_, and metabolic tumor volume (MTV) at 40% of SUV_max_ threshold (MTV_40_) were extracted for each VOI. No manual adjustment was added to the segmentation process.

To add the total metabolic tumor volume (TMTV) to the conventional parameters described above, a total-body PET segmentation tool (LesionID, version 7.0.5 of MIM Encore Software Inc., Cleveland, OH) was applied to each bPET/CT scan. As described in [30], the program workflow firstly used a PET Response Criteria in Solid Tumors (PERCIST)-based background threshold (liver) to identify all lesions with higher uptake, then applied a fixed relative threshold of ≥41% of the SUV_max_ of each VOI to create the boundaries of the metabolically active region within each lesion. Physicians were required to reject false-positive lesions (sites of physiological uptake, external contamination, and pathologic uptake deemed lymphoma-unrelated) before all approved VOIs were computed to obtain TMTV [30,31].

For the two target lesions, a rich set of additional radiomic features was extracted using Moddicom [32], an open-source software library in R [33] and Image Biomarker Standardization Initiative (IBSI)-compliant [34]. Moddicom’s image features belonged to the following IBSI classes: morphological, intensity-based statistical, intensity-histogram, grey-level co-occurrence matrix (GLCM), grey-level run-length matrix (GLRLM), and grey-level size-zone matrix (GLSZM) [34] (Supplemental Table S1). No spatial interpolation or kernel-based filter application to the images was needed before running them in the software due to the homogeneous geometry in the DICOM series.

### 2.4. Statistical Analysis and Radiomic Models

For early response-to-treatment assessment, complete metabolic response corresponded to iPET/CT DS 1–3, while DS 4–5 was associated to partial/no metabolic response [5]. Twenty-four-month PFS was defined as the interval between histological diagnosis of cHL and the first clinical detection of progression during treatment, treatment escalation, and lack of complete remission after PCT or disease relapse.

**Figure 2:**
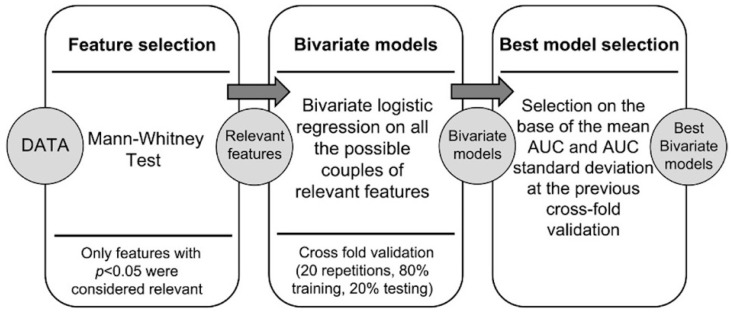
Radiomics computational pipeline.

Figure 2 shows the statistical workflow employed. Briefly, a Mann–Whitney test was performed to identify the most promising image features (*p* < 0.05) from Lesion_A and Lesion_B with regards to DS (<4 or ≥4) and 24-month PFS (“no event” or “event” at 24 months). Among the statistically significant features, the first 60 were used to build all possible bivariate models through logistic regression (LR) analysis. LR bivariate models were trained/tested with a cross-fold validation test (training set vs. testing set: 80% vs. 20%, 20 repetitions). The best models were then selected on the base of receiver operating characteristic (ROC) curves, mean area under the ROC curves (mAUC), and SD to the normal. Moreover, the same statistical workflow was applied to analyze radiomic data separately for each scanner (Scanner_1: Gemini GXL; Scanner_2: Biograph mCT).

## 3. Results

### 3.1. Patients’ Characteristics

**Figure 3:**
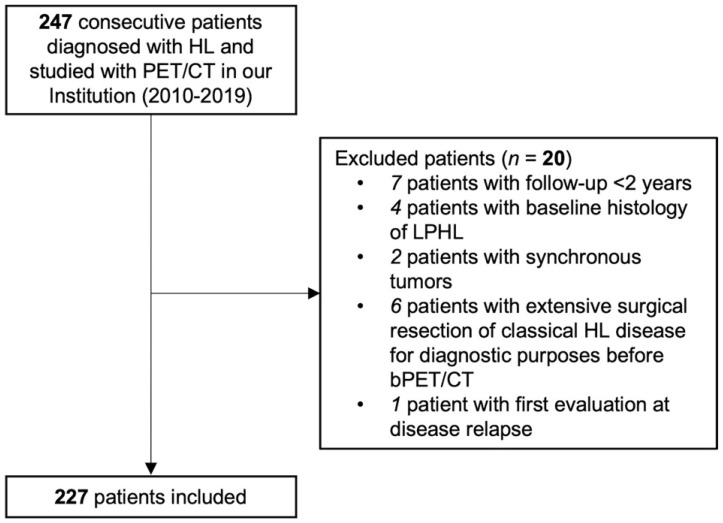
Flowchart of patients’ selection. HL, Hodgkin’s lymphoma; LPHL, nodular lymphocyte-predominant HL.

Among the 247 patients with cHL referred to the hematology unit between 2010 and 2019, 227 fulfilled the inclusion criteria and were included in the study (Figure 3). Patients’ characteristics are reported in Table 2. Disease stage was limited (I/II) in 51.5% (117/227) patients and advanced (stage III/IV) in 48.5% (110/227). Mean follow-up time was 56 months (range, 2–127). Adverse events at 2 years from bPET/CT were recorded in 46 patients. The 24-month PFS was 78.46%.

### 3.2. Radiomic Features

**Figure 4:**
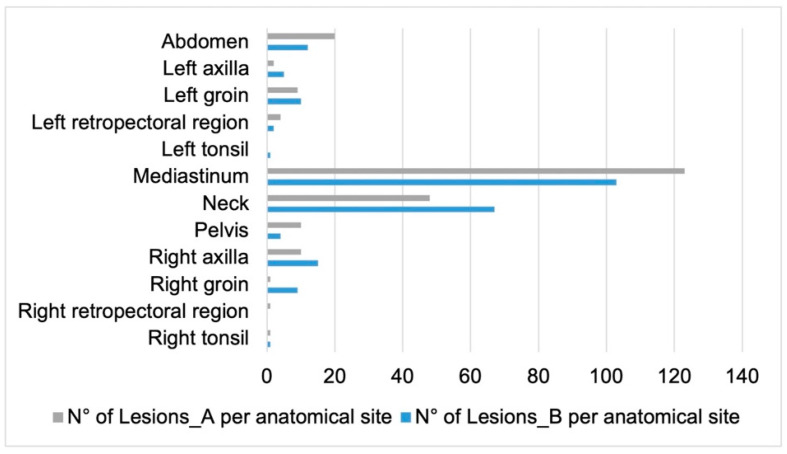
Anatomical site distribution of Lesion_A and Lesion_B among the entire cohort.

Table 3 shows bPET/CT conventional parameters extracted from Lesion_A and Lesion_B (D_max_, SUV_max_, SUV_mean_, and MTV_40_) and TMTV. Their statistical correlation with the outcomes is also itemized. For DS prediction, TMTV, Lesion_A_D_max_, and Lesion_A_MTV_40_ were among the first 60 significant features at Mann–Whitney test in the univariate phase of the radiomic computational pipeline (Figure 2). Among the first 60 most relevant features for 24-month PFS prediction, the conventional PET/CT parameters were Lesion_A_D_max_, Lesion_A_SUV_max_, Lesion_A_SUV_mean_, Lesion_A_MTV_40_, Lesion_B_D_max_, Lesion_B_SUV_max_, Lesion_B_SUV_mean_, Lesion_B_MTV_40_, and TMTV. Figure 4 shows the anatomical locations of Lesion_A and Lesion_B among all patients. The mediastinum was the most frequent site for both lesions (Figure 4).

At iPET/CT, DS was 1 for 31/227 patients (13.6%), 2 for 87/227 (38.3%), 3 for 63/227 (27.8%), 4 for 42/227 (18.5%), and 5 for 4/227 (1.8%). Image features extracted with Moddicom are described in Supplemental Table S1.

### 3.3. Radiomic Models

**Figure 5:**
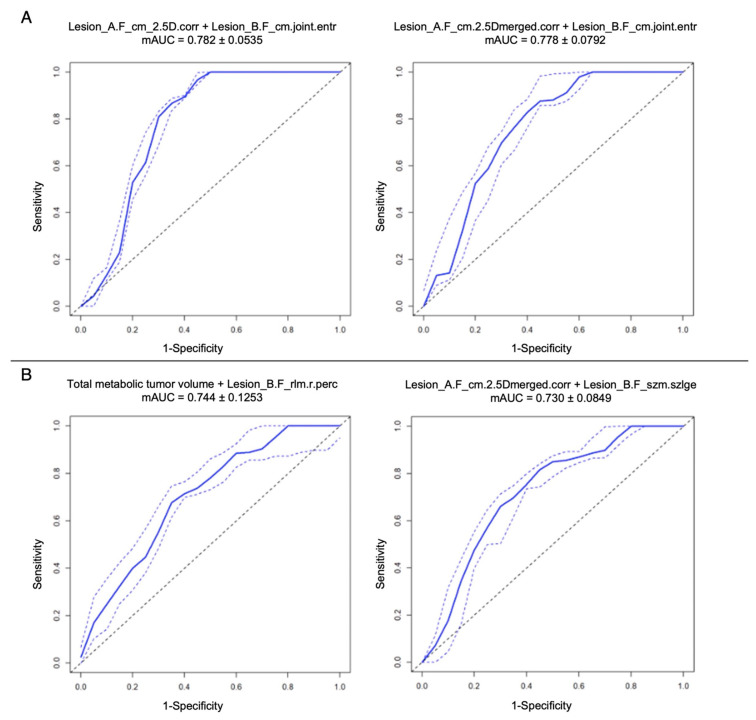
First two best bivariate radiomic models at cross-fold validation test for DS prediction (**A**) and for 24-month PFS prediction (**B**) in the overall cohort. The continuous blue line represents mAUC. The dotted blue lines represent +/− standard deviation.

The first two best bivariate models at cross-fold validation test for DS prediction are shown in Figure 5A, with a maximum mAUC of 0.78 ± 0.053 in the model combining a GLCM correlation feature from Lesion_A and the GLCM joint entropy feature from Lesion_B. Overall, Lesion_A concurred predominantly in the best bivariate models with features from several IBSI classes, while Lesion_B scantly contributed and almost only with the “entropy” feature from the GLCM class (Supplemental Table S2).

The first two best bivariate models for 24-month PFS prediction are shown in Figure 5B, with the maximum mAUC (0.74 ± 0.12) found in the combination of TMTV and a Lesion_B GLRLM feature. In this case, the overall best combinations saw no contribution from Lesion_A radiomic features. Lesion_B instead, especially combined with TMTV, was the most representative, with features belonging to numerous IBSI classes (Supplemental Table S3).

### 3.4. Scanner-Based Radiomic Models

**Figure 6:**
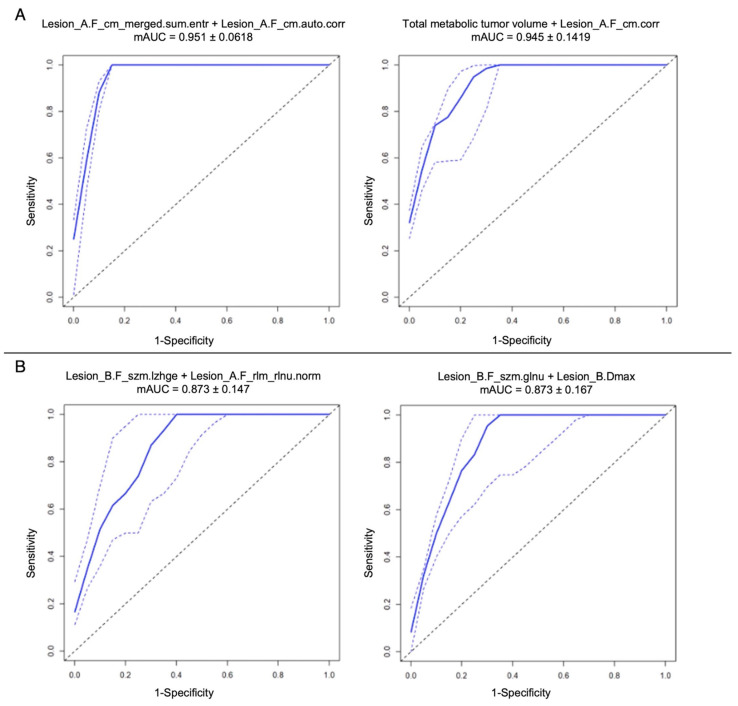
First two best bivariate radiomic models at cross-fold validation test for DS prediction with data obtained from Scanner_1 images (**A**) and for 24-month PFS prediction with data obtained from Scanner_2 images (**B**). The continuous blue line represents mAUC. The dotted blue lines represent +/− standard deviation.

At bPET/CT, 119/227 patients (52.4%) were scanned on Scanner_1 and 108/227 (47.6%) on Scanner_2. For DS prediction, Scanner_1 was the best-performing for every relevant feature. The best bivariate radiomic models came from the combination of Lesion_A features belonging to different IBSI classes, more often GLCM-related ones (Supplemental Table S4), with a minor contribution of TMTV. The best mAUC (0.95 ± 0.06) was obtained combining Lesion_A entropy and autocorrelation features from GLCM class (Figure 6A). The best bivariate radiomic models for 24-month PFS prediction were found with features extracted from Scanner_2 images (Figure 6B, maximum mAUC: 0.87 ± 0.14), with features almost all belonging to Lesion_B (Supplemental Table S5).

## 4. Discussion

This study shows that bivariate models of bPET/CT radiomic features primarily from the largest lesion (Lesion_A) likely foresee cHL patients’ response to early evaluation during PCT (DS at iPET/CT), while bivariate models of radiomic features primarily from the hottest lesion (Lesion_B) seem to predict patients’ long-term outcome (PFS). The dichotomy became even more evident, and radiomic models also had higher prognostic power when analyzing image data divided by scanner. Conceivably, there may be a different grade of strength in how a feature can provide informative contents with regards to a specific outcome depending on the underlying scanner technology. Indeed, bivariate models of mainly morphology-related radiomic features belonging to Lesion_A (the largest) had higher significance for DS prediction when extracted from Scanner_1 (Philips Gemini GXL). A possible explanation is that the morphology of the lesions might have been influenced by PSF modeling, TOF, and image-smoothing applied during image reconstruction on Scanner_2 (Siemens Biograph mCT) but not on Scanner_1. Instead, for PFS prediction, bivariate analysis was successful on features belonging to Lesion_B (the hottest) and on Scanner_2. Interestingly, the best models often combined features from GLSZM and GLRLM classes, which deal with grey-level discretization [34]. Scanner_2 has a more up-to-date technology compared to Scanner_1, and its higher spatial resolution may explain our results.

Besides the two target lesions, significant prognostic power was also provided by TMTV for both DS and PFS. This finding is in line with other studies [35,36]. However, an applicable threshold has not currently been identified for TMTV as for other radiomic features, and calculation methodology has varied among studies [12,13,37,38], preventing its use in clinical routine settings [13,16]. In our study, a gradient-based algorithm for target volume segmentation was employed, considering its known higher accuracy and consistency compared to constant-threshold or manual contouring (especially for lesions <2 cm) [28,39]. TMTV instead was assessed using a fixed relative threshold method [37], which in a single automatic program workflow has been previously described [38,40,41] and deemed to allow fast, reproducible, and practical calculations in patients with disseminated disease [38]. However, recent investigations using different scanners and segmentation methods in both HL and non-HL have interestingly concluded that calculations from different contouring techniques generate similar results [38,42,43].

To our knowledge, this is the first study in the literature proposing a reasonable trade-off between previously suggested numbers of target lesions and methodological approaches, and we believe it could draw a feasible path towards application in clinical practice. Previous reports have described correlation between tumoral bulks and DS [19,44], high SUV_max_ and tumor aggressiveness [45,46,47], radiomics from one/several/all lymphoma lesions, and prognosis in univariate/multivariate analyses, though mostly in small cohorts and with no model validation [12,13].

However, the present study has some limitations. The first is its retrospective, single-center nature, which influenced population numerosity. Even if quite a numerous cohort was recruited compared to other cHL studies in the literature, we recognize it is still limited for our results to be generalizable. Moreover, this characteristic limited the possibility of performing per-stage and per-histological subtype sub-analyses. On the one hand, the heterogeneity of our population may be seen as representative of a real-life clinical scenario and inclusion-bias avoidance, with proportions of the distribution of disease stage percentages matching the ones of the US/EU population, with stage II being the more frequently presented at diagnosis, followed by stages IV, III, and I. On the other hand, histology and stage at diagnosis are known to impact patients’ response to therapy and survival [1,2,4,5,6] and will therefore need to be taken into account in further larger studies. We also limited our analysis to PET parameters, overlooking CT parameters other than D_max_ [17,48]. All volumes corresponding to the chosen criteria for radiomic target lesions were analyzed regardless of size, but the use of an IBSI-compliant platform that succeeded analyzing small volumes may be rather seen as an overcoming of what was a challenge in other studies [43]. The use of images acquired from different scanners and the lack of resampling or harmonization of the acquired images may be considered as further limitations [49]. Conversely, these traits combined with avoidance of manual adjustments to the image contouring method proposed may allow time saving, consistency, and generalization of our results to other institutions for a desirable external validation, which would help in strengthening and anticipating the decision-making process in cHL patients’ treatment by combining the prognostic power of two target lesions at bPET/CT. Multicenter studies with a larger and more homogeneous cohort may allow for stronger results and comparison with conventional clinical prognostic models.

## 5. Conclusions

This large monocentric retrospective study on cHL patients offers a broader insight into baseline PET/CT and shows that bivariate radiomic models from the largest and the hottest lesions provide significant information about patients’ outcome (DS in iPET/CT and PFS, respectively). TMTV has prognostic significance for both DS and PFS. Bivariate models of higher prognostic power were found when the underlying scanner technology was considered, unveiling possible image morphological distortions following appliance of multiple reconstruction algorithms.

Further studies including correlations with clinical parameters and external validation of our proposed model are auspicial.

## Figures and Tables

**Table 1:** Acquisition settings of the two employed scanners.

	Acquisition Protocol	Scanner_1 Philips Gemini GXL	Scanner_2 Siemens Biograph mCT
	kV	120	
	mAs	40–50	
CT	Image matrix size	512 × 512	
	Voxel size (mm)	3.5 × 3.5 × 5	0.97 × 0.97 × 3
	Field of view	From skull base to mid-thighs	
	Reconstruction method	3D-LOR-RAMLA No PSF No TOF	3D-OSEM PSF TOF
	Iterations	3	2
	Subsets	33	21
PET	Voxel size (mm)	4 × 4 × 4	3.2 × 3.2 × 5
	Filters applied	Gaussian filter 5 mm	Gaussian filter 2 mm
	Axial matrix size	128 × 128	400 × 400
	Minutes per bed position	3	2.5
	Field-of-view	From skull base to mid-thighs	

LOR-RAMLA, line of response-row-action maximum likelihood algorithm; 3D-OSEM, ordered subset expectation maximization; PSF, point-spread-function; TOF, time of flight.

**Table 2:** Patients’ characteristics (*n* = 227).

Sex: *n* (%)	
Male	110 (48.5%)
Female	117 (51.5%)
Age at diagnosis: years	
Mean (Range)	40 (16–83)
Ann Arbor Stage: *n* (%)	
I	3 (1.3%)
II	114 (50.2%)
III	38 (16.8%)
IV	72 (31.7%)
Bulk: *n* (%)	
Yes	45 (19.8%)
No	182 (80.2%)
Disease sites: *n* (%)	
Only nodal	117 (51.5%)
Nodal and extranodal	110 (48.5%)
Histological subtype: *n* (%)	
Nodular sclerosing	174 (76.6%)
Mixed cellularity	12 (5.3%)
Lymphocyte-rich	6 (2.7%)
Lymphocyte-depleted	5 (2.2%)
cHL-NOS	30 (13.2%)
PCT: *n* (%)	
ABVD/MBVD	208 (91.6%)
BEACOPP	13 (5.7%)
Other	6 (2.7%)
Post-PCT radiotherapy on involved field	185 (81.5%)
Therapy escalation during PCT	22 (9.7%)

cHL-NOS, classical Hodgkin’s lymphoma with no possible further histological subtype assessment; PCT, primary chemotherapy.

**Table 3:** Characteristics of bPET/CT conventional parameters and their correlation with outcomes (DS and 24-month PFS).

PET/CT Parameters	Lesion_A	Lesion_B
Dmax (cm)		
Mean	4.45	2.09
Median	9	1
Range	0.58–11.13	0.34–6.43
DS-corr (p)	0.001 *	0.483
PFS-corr (p)	0.009 *	0.019 *
SUVmax		
Mean	14.44	13.41
Median	21	11.5
Range	2.55–66	2.19–39.13
DS-corr (p)	0.032 *	0.062
PFS-corr (p)	0.011 *	0.021 *
SUVmean		
Mean	6.7	8
Median	8	14
Range	1.28–22.14	1.5–27.68
DS-corr (p)	0.299	0.235
PFS-corr (p)	0.012 *	0.021 *
MTV_40_ (mL)		
Mean	77.97	9.08
Median	55	4
Range	0.1–721.16	0.02–139.19
DS-corr (p)	0.001 *	0.485
PFS-corr (p)	0.017 *	0.025 *
TMTV (mL)		
Mean	237.77	
Median	331.5	
Range	0.73–1145.68	
DS-corr (p)	0.0005 *	
PFS-corr (p)	0.003 *	

Dmax, maximal axial diameter; DS-corr, *p*-value of the correlation coefficient with Deauville score; PFS-corr, *p*-value of the correlation coefficient with 24-month progression-free survival; SUV, standard uptake value; MTV, metabolic tumor volume; TMTV, total metabolic tumor volume; *, *p*-value < 0.05.

## Data Availability

The data presented in this study are available on request from the corresponding author.

## Supplementary Materials

The following supporting information can be downloaded at: https://www.mdpi.com/article/10.3390/diagnostics13081391/s1, Table S1: Radiomic features classes and their components, extracted from Lesion_A and Lesion_B through Moddicom software; Table S2: Best bivariate radiomic models for Deauville score prediction (whole cohort); Table S3: Best bivariate radiomic models for 24-month progression-free survival prediction (whole cohort); Table S4: Best bivariate radiomic models for Deauville score prediction (Scanner_1 images); Table S5: Best bivariate models for 24-month progression-free survival prediction (Scanner_2 images).

## References

[1] Eichenauer D.A., Aleman B.M.P., André M., Federico M., Hutchings M., Illidge T., Engert A., Ladetto M. (2018). Hodgkin Lymphoma: ESMO Clinical Practice Guidelines for Diagnosis, Treatment and Follow-Up. Ann. Oncol..

[2] SEER*Explorer: An Interactive Website for SEER Cancer Statistics. https://Seer.Cancer.Gov/Explorer/.

[3] Ansell S.M. (2020). Hodgkin Lymphoma: A 2020 Update on Diagnosis, Risk-stratification, and Management. Am. J. Hematol..

[4] Barrington S.F., Mikhaeel N.G., Kostakoglu L., Meignan M., Hutchings M., Müeller S.P., Schwartz L.H., Zucca E., Fisher R.I., Trotman J. (2014). Role of Imaging in the Staging and Response Assessment of Lymphoma: Consensus of the International Conference on Malignant Lymphomas Imaging Working Group. J. Clin. Oncol..

[5] Cheson B.D., Fisher R.I., Barrington S.F., Cavalli F., Schwartz L.H., Zucca E., Lister T.A. (2014). Recommendations for Initial Evaluation, Staging, and Response Assessment of Hodgkin and Non-Hodgkin Lymphoma: The Lugano Classification. J. Clin. Oncol..

[6] Hoppe R.T., Advani R.H., Ai W.Z., Ambinder R.F., Armand P., Bello C.M., Benitez C.M., Bierman P.J., Boughan K.M., Dabaja B. (2022). Hodgkin Lymphoma, Version 2.2022, NCCN Clinical Practice Guidelines in Oncology. J. Natl. Compr. Cancer Netw..

[7] Spinner M.A., Advani R.H. (2018). Risk-Adapted Therapy for Advanced-Stage Hodgkin Lymphoma. Hematology.

[8] Meignan M., Gallamini A., Meignan M., Gallamini A., Haioun C. (2009). Report on the First International Workshop on Interim-PET Scan in Lymphoma. Leuk. Lymphoma.

[9] Barrington S.F., Qian W., Somer E.J., Franceschetto A., Bagni B., Brun E., Almquist H., Loft A., Højgaard L., Federico M. (2010). Concordance between Four European Centres of PET Reporting Criteria Designed for Use in Multicentre Trials in Hodgkin Lymphoma. Eur. J. Nucl. Med. Mol. Imaging.

[10] Annunziata S., Cuccaro A., Calcagni M.L., Hohaus S., Giordano A., Rufini V. (2016). Interim FDG-PET/CT in Hodgkin Lymphoma: The Prognostic Role of the Ratio between Target Lesion and Liver SUVmax (RPET). Ann. Nucl. Med..

[11] Engert A., Diehl V., Franklin J., Lohri A., Dörken B., Ludwig W.-D., Koch P., Hänel M., Pfreundschuh M., Wilhelm M. (2009). Escalated-Dose BEACOPP in the Treatment of Patients With Advanced-Stage Hodgkin’s Lymphoma: 10 Years of Follow-Up of the GHSG HD9 Study. J. Clin. Oncol..

[12] Wang H., Zhou Y., Li L., Hou W., Ma X., Tian R. (2020). Current Status and Quality of Radiomics Studies in Lymphoma: A Systematic Review. Eur. Radiol..

[13] Rizzo A., Triumbari E.K.A., Gatta R., Boldrini L., Racca M., Mayerhoefer M., Annunziata S. (2021). The Role of 18F-FDG PET/CT Radiomics in Lymphoma. Clin. Transl. Imaging.

[14] Frood R., Burton C., Tsoumpas C., Frangi A.F., Gleeson F., Patel C., Scarsbrook A. (2021). Baseline PET/CT Imaging Parameters for Prediction of Treatment Outcome in Hodgkin and Diffuse Large B Cell Lymphoma: A Systematic Review. Eur. J. Nucl. Med. Mol. Imaging.

[15] Morland D., Triumbari E.K.A., Boldrini L., Gatta R., Pizzuto D., Annunziata S. (2022). Radiomics in Oncological PET Imaging: A Systematic Review—Part 2, Infradiaphragmatic Cancers, Blood Malignancies, Melanoma and Musculoskeletal Cancers. Diagnostics.

[16] Lippi M., Gianotti S., Fama A., Casali M., Barbolini E., Ferrari A., Fioroni F., Iori M., Luminari S., Menga M. (2020). Texture Analysis and Multiple-Instance Learning for the Classification of Malignant Lymphomas. Comput. Methods Programs Biomed..

[17] Lartizien C., Rogez M., Niaf E., Ricard F. (2014). Computer-Aided Staging of Lymphoma Patients With FDG PET/CT Imaging Based on Textural Information. IEEE J. Biomed. Health Inform..

[18] Kenawy M.A., Khalil M.M., Abdelgawad M.H., El-Bahnasawy H.H. (2020). Correlation of Texture Feature Analysis with Bone Marrow Infiltration in Initial Staging of Patients with Lymphoma Using 18F-Fluorodeoxyglucose Positron Emission Tomography Combined with Computed Tomography. Pol. J. Radiol..

[19] Ben Bouallègue F., Tabaa Y.A., Kafrouni M., Cartron G., Vauchot F., Mariano-Goulart D. (2017). Association between Textural and Morphological Tumor Indices on Baseline PET-CT and Early Metabolic Response on Interim PET-CT in Bulky Malignant Lymphomas. Med. Phys..

[20] Lue K.-H., Wu Y.-F., Liu S.-H., Hsieh T.-C., Chuang K.-S., Lin H.-H., Chen Y.-H. (2020). Intratumor Heterogeneity Assessed by 18F-FDG PET/CT Predicts Treatment Response and Survival Outcomes in Patients with Hodgkin Lymphoma. Acad. Radiol..

[21] Rodríguez Taroco M.G., Cuña E.G., Pages C., Schelotto M., González-Sprinberg G.A., Castillo L.A., Alonso O. (2021). Prognostic Value of Imaging Markers from 18FDG-PET/CT in Paediatric Patients with Hodgkin Lymphoma. Nucl. Med. Commun..

[22] Lue K.-H., Wu Y.-F., Liu S.-H., Hsieh T.-C., Chuang K.-S., Lin H.-H., Chen Y.-H. (2019). Prognostic Value of Pretreatment Radiomic Features of 18F-FDG PET in Patients With Hodgkin Lymphoma. Clin. Nucl. Med..

[23] Milgrom S.A., Elhalawani H., Lee J., Wang Q., Mohamed A.S.R., Dabaja B.S., Pinnix C.C., Gunther J.R., Court L., Rao A. (2019). A PET Radiomics Model to Predict Refractory Mediastinal Hodgkin Lymphoma. Sci. Rep..

[24] Sollini M., Kirienko M., Cavinato L., Ricci F., Biroli M., Ieva F., Calderoni L., Tabacchi E., Nanni C., Zinzani P.L. (2020). Methodological Framework for Radiomics Applications in Hodgkin’s Lymphoma. Eur. J. Hybrid Imaging.

[25] Zwanenburg A. (2019). Radiomics in Nuclear Medicine: Robustness, Reproducibility, Standardization, and How to Avoid Data Analysis Traps and Replication Crisis. Eur. J. Nucl. Med. Mol. Imaging.

[26] Driessen J., Zwezerijnen G.J., Schöder H., Drees E.E., Kersten M.J., Moskowitz A.J., Moskowitz C.H., Eertink J.J., De Vet H.C., Hoekstra O.S. (2022). The Impact of Semi-Automatic Segmentation Methods on Metabolic Tumor Volume, Intensity and Dissemination Radiomics in ^18^F-FDG PET Scans of Patients with Classical Hodgkin Lymphoma. J. Nucl. Med..

[27] Boellaard R., Delgado-Bolton R., Oyen W.J.G., Giammarile F., Tatsch K., Eschner W., Verzijlbergen F.J., Barrington S.F., Pike L.C., Weber W.A. (2015). FDG PET/CT: EANM Procedure Guidelines for Tumour Imaging: Version 2.0. Eur. J. Nucl. Med. Mol. Imaging.

[28] Werner-Wasik M., Nelson A.D., Choi W., Arai Y., Faulhaber P.F., Kang P., Almeida F.D., Xiao Y., Ohri N., Brockway K.D. (2012). What Is the Best Way to Contour Lung Tumors on PET Scans? Multiobserver Validation of a Gradient-Based Method Using a NSCLC Digital PET Phantom. Int. J. Radiat. Oncol. Biol. Phys..

[29] Dibble E.H., Lara Alvarez A.C., Truong M.-T., Mercier G., Cook E.F., Subramaniam R.M. (2012). ^18^F-FDG Metabolic Tumor Volume and Total Glycolytic Activity of Oral Cavity and Oropharyngeal Squamous Cell Cancer: Adding Value to Clinical Staging. J. Nucl. Med..

[30] Dean E.A., Mhaskar R.S., Lu H., Mousa M.S., Krivenko G.S., Lazaryan A., Bachmeier C.A., Chavez J.C., Nishihori T., Davila M.L. (2020). High Metabolic Tumor Volume Is Associated with Decreased Efficacy of Axicabtagene Ciloleucel in Large B-Cell Lymphoma. Blood Adv..

[31] Triumbari E.K.A., Morland D., Cuccaro A., Maiolo E., Hohaus S., Annunziata S. (2022). Classical Hodgkin Lymphoma: A Joint Clinical and PET Model to Predict Poor Responders at Interim Assessment. Diagnostics.

[32] Dinapoli N., Alitto A.R., Vallati M., Gatta R., Autorino R., Boldrini L., Damiani A., Valentini V. (2015). Moddicom: A Complete and Easily Accessible Library for Prognostic Evaluations Relying on Image Features. Proceedings of the 2015 37th Annual International Conference of the IEEE Engineering in Medicine and Biology Society (EMBC).

[33] R Core Team (2020). R: A Language and Environment for Statistical Computing.

[34] Zwanenburg A., Vallières M., Abdalah M.A., Aerts H.J.W.L., Andrearczyk V., Apte A., Ashrafinia S., Bakas S., Beukinga R.J., Boellaard R. (2020). The Image Biomarker Standardization Initiative: Standardized Quantitative Radiomics for High-Throughput Image-Based Phenotyping. Radiology.

[35] Feres C.C.P., Nunes R.F., Teixeira L.L.C., Arcuri L.J., Perini G.F. (2022). Baseline Total Metabolic Tumor Volume (TMTV) Application in Hodgkin Lymphoma: A Review Article. Clin. Transl. Imaging.

[36] Zaucha J.M., Chauvie S., Zaucha R., Biggii A., Gallamini A. (2019). The Role of PET/CT in the Modern Treatment of Hodgkin Lymphoma. Cancer Treat. Rev..

[37] Im H.-J., Bradshaw T., Solaiyappan M., Cho S.Y. (2018). Current Methods to Define Metabolic Tumor Volume in Positron Emission Tomography: Which One Is Better?. Nucl. Med. Mol. Imaging.

[38] Camacho M.R., Etchebehere E., Tardelli N., Delamain M.T., Vercosa A.F.A., Takahashi M.E.S., Brunetto S.Q., Metze I.G.H.L., Souza C.A., Cerci J.J. (2020). Validation of a Multifocal Segmentation Method for Measuring Metabolic Tumor Volume in Hodgkin Lymphoma. J. Nucl. Med. Technol..

[39] Wanet M., Lee J.A., Weynand B., De Bast M., Poncelet A., Lacroix V., Coche E., Grégoire V., Geets X. (2011). Gradient-Based Delineation of the Primary GTV on FDG-PET in Non-Small Cell Lung Cancer: A Comparison with Threshold-Based Approaches, CT and Surgical Specimens. Radiother. Oncol..

[40] Niman R., Buteau J.P., Kruzer A., Turcotte Ã., Nelson A. (2018). Evaluation of a Semi-Automated Whole Body PET Segmentation Method Applied to Diffuse Large B Cell Lymphoma. J. Nucl. Med..

[41] Damsky W., Wang A., Young B.D., Ayasun R., Ryu C., McGeary M.K., Fazzone-Chettiar R., Pucar D., Gulati M., Miller E.J. (2021). Treatment of Sarcoidosis with Cutaneous Involvement with Tofacitinib. medRixv.

[42] Kirienko M., Cozzi L., Antunovic L., Lozza L., Fogliata A., Voulaz E., Rossi A., Chiti A., Sollini M. (2018). Prediction of Disease-Free Survival by the PET/CT Radiomic Signature in Non-Small Cell Lung Cancer Patients Undergoing Surgery. Eur. J. Nucl. Med. Mol. Imaging.

[43] Eertink J.J., Pfaehler E.A.G., Wiegers S.E., van de Brug T., Lugtenburg P.J., Hoekstra O.S., Zijlstra J.M., de Vet H.C.W., Boellaard R. (2022). Quantitative Radiomics Features in Diffuse Large B-Cell Lymphoma: Does Segmentation Method Matter?. J. Nucl. Med..

[44] Lopez-Alonso R., Qi S., Mashiach T., Weiler-Sagie M., Yahalom J., Dann E.J. (2021). The Presence of a Bulky Mediastinal Mass of 7 Cm or Greater in Diameter Confers an Adverse Prognosis to Patients with Advanced Hodgkin Lymphoma in Case of Negative Interim PET/CT. Leuk. Lymphoma.

[45] Xie M., Wu K., Liu Y., Jiang Q., Xie Y. (2015). Predictive Value of F-18 FDG PET/CT Quantization Parameters in Diffuse Large B Cell Lymphoma: A Meta-Analysis with 702 Participants. Med. Oncol..

[46] Tatsumi M., Isohashi K., Matsunaga K., Watabe T., Kato H., Kanakura Y., Hatazawa J. (2019). Volumetric and Texture Analysis on FDG PET in Evaluating and Predicting Treatment Response and Recurrence after Chemotherapy in Follicular Lymphoma. Int J. Clin. Oncol..

[47] Xia X., Wang Y., Yuan J., Sun W., Jiang J., Liu C., Zhang Q., Ma X. (2020). Baseline SUVmax of 18F-FDG PET-CT Indicates Prognosis of Extranodal Natural Killer/T-Cell Lymphoma. Medicine.

[48] Ganeshan B., Miles K.A., Babikir S., Shortman R., Afaq A., Ardeshna K.M., Groves A.M., Kayani I. (2017). CT-Based Texture Analysis Potentially Provides Prognostic Information Complementary to Interim Fdg-Pet for Patients with Hodgkin’s and Aggressive Non-Hodgkin’s Lymphomas. Eur. Radiol..

[49] Mayerhoefer M.E., Materka A., Langs G., Häggström I., Szczypiński P., Gibbs P., Cook G. (2020). Introduction to Radiomics. J. Nucl. Med..

