# Supplementary material for: Baseline 18F-FDG PET/CT Radiomics in Classical Hodgkin’s Lymphoma: The Predictive Role of the Largest and the Hottest Lesions
Source: Diagnostics (Basel). 2023 Apr 11;13(8):1391. doi: 10.3390/diagnostics13081391 (PMC10137254; doi:10.3390/diagnostics13081391)
Supplement: Supplementary file 1 [file diagnostics-13-01391-s001.zip › Triumbari_Supplemental_Table S1.pdf]

SUPPLEMENTAL TABLE S1. Radiomic features classes and their components, extracted from Lesion_A and Lesion_B through Moddicom software

IBSI* Class	Features			
Aggregation method	*Averaged*	*2.5 D (direction merged)*	*Merged*	*2.5 D, Merged*
**Morphological**	F_morph.surface			
	F_morph.volume			
	F_morph.av			
	F_morph.comp.1			
	F_morph.comp.2			
	F_morph.sph.dispr			
	F_morph.sphericity			
	F_morph.asphericity			
	F_morph.com			
	F_morph.pca.major			
	F_morph.pca.minor			
	F_morph.pca.least			
	F_morph.pca.elongation			
	F_morph.pca.flatness			
**Intensity-based statistical**	F_stat.mean			
	F_stat.var			
	F_stat.skew			
	F_stat.kurt			
	F_stat.median			
	F_stat.min			
	F_stat.10thpercentile			
	F_stat.90thpercentile			
	F_stat.max			
	F_stat.iqr			
	F_stat.range			
	F_stat.mad			
	F_stat.rmad			
	F_stat.energy			
	F_stat.rms			
**Intensity-histogram**	F_stat.entropy			
	F_stat.uniformity			
**GLCM**[†]	F_cm.joint.max	F_cm_2.5D.joint.max	F_cm_merged.joint.max	F_cm.2.5Dmerged.joint.max
Presents the number of times that two intensity levels have occurred in two pixels with specific distance	F_cm.joint.avg	F_cm_2.5D.joint.avg	F_cm_merged.joint.avg	F_cm.2.5Dmerged.joint.avg
	F_cm.joint.var	F_cm_2.5D.joint.var	F_cm_merged.joint.var	F_cm.2.5Dmerged.joint.var
	F_cm.joint.entr	F_cm_2.5D.joint.entr	F_cm_merged.joint.entr	F_cm.2.5Dmerged.joint.entr
	F_cm.diff.avg	F_cm_2.5D.diff.avg	F_cm_merged.diff.avg	F_cm.2.5Dmerged.diff.avg
	F_cm.diff.var	F_cm_2.5D.diff.var	F_cm_merged.diff.var	F_cm.2.5Dmerged.diff.var
	F_cm.diff.entr	F_cm_2.5D.diff.entr	F_cm_merged.diff.entr	F_cm.2.5Dmerged.diff.entr
	F_cm.sum.avg	F_cm_2.5D.sum.avg	F_cm_merged.sum.avg	F_cm.2.5Dmerged.sum.avg
	F_cm.sum.var	F_cm_2.5D.sum.var	F_cm_merged.sum.var	F_cm.2.5Dmerged.sum.var
	F_cm.sum.entr	F_cm_2.5D.sum.entr	F_cm_merged.sum.entr	F_cm.2.5Dmerged.sum.entr
	F_cm.energy	F_cm_2.5D.energy	F_cm_merged.energy	F_cm.2.5Dmerged.energy
	F_cm.contrast	F_cm_2.5D.contrast	F_cm_merged.contrast	F_cm.2.5Dmerged.contrast
	F_cm.dissimilarity	F_cm_2.5D.dissimilarity	F_cm_merged.dissimilarity	F_cm.2.5Dmerged.dissimilarity
	F_cm.inv.diff	F_cm_2.5D.inv.diff	F_cm_merged.inv.diff	F_cm.2.5Dmerged.inv.diff
	F_cm.inv.diff.norm	F_cm_2.5D.inv.diff.norm	F_cm_merged.inv.diff.norm	F_cm.2.5Dmerged.inv.diff.norm
	F_cm.inv.diff.mom	F_cm_2.5D.inv.diff.mom	F_cm_merged.inv.diff.mom	F_cm.2.5Dmerged.inv.diff.mom
	F_cm.inv.diff.mom.norm	F_cm_2.5D.inv.diff.mom.norm	F_cm_merged.inv.diff.mom.norm	F_cm.2.5Dmerged.inv.diff.mom.norm
	F_cm.inv.var	F_cm_2.5D.inv.var	F_cm_merged.inv.var	F_cm.2.5Dmerged.inv.var
	F_cm.corr	F_cm_2.5D.corr	F_cm_merged.corr	F_cm.2.5Dmerged.corr
	F_cm.auto.corr	F_cm_2.5D.auto.corr	F_cm_merged.auto.corr	F_cm.2.5Dmerged.auto.corr
	F_cm.clust.tend	F_cm_2.5D.clust.tend	F_cm_merged.clust.tend	F_cm.2.5Dmerged.clust.tend
	F_cm.clust.shade	F_cm_2.5D.clust.shade	F_cm_merged.clust.shade	F_cm.2.5Dmerged.clust.shade
	F_cm.clust.prom	F_cm_2.5D.clust.prom	F_cm_merged.clust.prom	F_cm.2.5Dmerged.clust.prom
	F_cm.info.corr.1	F_cm_2.5D.info.corr.1	F_cm_merged.info.corr.1	F_cm.2.5Dmerged.info.corr.1
	F_cm.info.corr.2	F_cm_2.5D.info.corr.2	F_cm_merged.info.corr.2	F_cm.2.5Dmerged.info.corr.2
**GLRLM**[‡]	F_rlm.sre	F_rlm_2.5D.sre		F_rlm.2.5Dmerged.sre
Presents the length of consecutive pixels having the same intensity	F_rlm.lre	F_rlm_2.5D.lre		F_rlm.2.5Dmerged.lre
	F_rlm.lgre	F_rlm_2.5D.lgre		F_rlm.2.5Dmerged.lgre
	F_rlm.hgre	F_rlm_2.5D.hgre		F_rlm.2.5Dmerged.hgre
	F_rlm.srlge	F_rlm_2.5D.srlge		F_rlm.2.5Dmerged.srlge
	F_rlm.srhge	F_rlm_2.5D.srhge		F_rlm.2.5Dmerged.srhge
	F_rlm.lrlge	F_rlm_2.5D.lrlge		F_rlm.2.5Dmerged.lrlge
	F_rlm.lrhge	F_rlm_2.5D.lrhge		F_rlm.2.5Dmerged.lrhge
	F_rlm.glnu	F_rlm_2.5D.glnu		F_rlm.2.5Dmerged.glnu

	F_rlm.glnu.norm<br>F_rlm.rlnu<br>F_rlm.rlnu.norm<br>F_rlm.r.perc<br>F_rlm.gl.var<br>F_rlm.rl.var<br>F_rlm.rl.entr	F_rlm_2.5D.glnu.norm<br>F_rlm_2.5D.rlnu<br>F_rlm_2.5D.rlnu.norm<br>F_rlm_2.5D.r.perc<br>F_rlm_2.5D.gl.var<br>F_rlm_2.5D.rl.var<br>F_rlm_2.5D.rl.entr<br>F_rlm_25D_merged.dfge		F_rlm.2.5Dmerged.glnu.norm<br>F_rlm.2.5Dmerged.rlnu<br>F_rlm.2.5Dmerged.rlnu.norm<br>F_rlm.2.5Dmerged.r.perc<br>F_rlm.2.5Dmerged.gl.var<br>F_rlm.2.5Dmerged.rl.var<br>F_rlm.2.5Dmerged.rl.entr
**GLSZM§**<br>Considers the size of homogeneous zones in every dimension	F_szm.sze<br>F_szm.lze<br>F_szm.lgze<br>F_szm.hgze<br>F_szm.szlge<br>F_szm.szhge<br>F_szm.lzlge<br>F_szm.lzhge<br>F_szm.glnu<br>F_szm.glnu.norm<br>F_szm.zsnu,<br>F_szm.zsnu.norm<br>F_zsm.z.perc<br>F_szm.gl.var<br>F_szm.zs.var<br>F_szm.z.entr	F_szm_2.5D.sze<br>F_szm_2.5D.lze<br>F_szm_2.5D.lgze<br>F_szm_2.5D.hgze<br>F_szm_2.5D.szlge<br>F_szm_2.5D.szhge<br>F_szm_2.5D.lzlge<br>F_szm_2.5D.lzhge<br>F_szm_2.5D.glnu<br>F_szm_2.5D.glnu.norm<br>F_szm_2.5D.zsnu<br>F_szm_2.5D.zsnu.norm<br>F_zsm_2.5D.z.perc<br>F_szm_2.5D.gl.var<br>F_szm_2.5D.zs.var<br>F_szm_2.5D.z.entr		

*: Image biomarker standardization initiative
†: Grey Level Co-occurrence Matrix-based features.
‡: Grey Level Run Length Matrix-based features.
§: Grey Level Size Zone Matrix-based features.
