# Supplementary material for: Baseline 18F-FDG PET/CT Radiomics in Classical Hodgkin’s Lymphoma: The Predictive Role of the Largest and the Hottest Lesions
Source: Diagnostics (Basel). 2023 Apr 11;13(8):1391. doi: 10.3390/diagnostics13081391 (PMC10137254; doi:10.3390/diagnostics13081391)
Supplement: Supplementary file 1 [file diagnostics-13-01391-s001.zip › Triumbari_Supplemental_Table S2.pdf]

SUPPLEMENTAL TABLE S2. Best bivariate radiomic models for Deauville Score prediction (whole cohort)

**Covariate 1**	**Covariate 2**	**mAUC***	**SD† AUC**
Lesion_A.F_cm_2.5D.corr	Lesion_B.F_cm.joint.entr	0.782	0.0535
Lesion_A.F_cm.2.5Dmerged.corr	Lesion_B.F_cm.joint.entr	0.778	0.0792
Lesion_A.F_szm_2.5D.lze	Lesion_B.F_cm.joint.entr	0.773	0.1103
Lesion_A.F_rlm.glnu	Lesion_B.F_cm.joint.entr	0.772	0.1166
Lesion_A.F_rlm.lre	Lesion_B.F_cm_2.5D.corr	0.770	0.0811
Lesion_A.F_rlm.rlnu	Lesion_A.F_stat.uniformity	0.769	0.1395
TMTV‡	Lesion_A.F_rlm.lgre	0.763	0.0849
TMTV	Lesion_A.F_morph.surface	0.761	0.0979
TMTV	Lesion_B.F_cm_2.5D.joint.entr	0.756	0.0909
Lesion_A.F_cm_2.5D.corr	Lesion_A.F_rlm.2.5Dmerged.rlnu	0.752	0.0454

* Mean area under the ROC curve.

† Standard deviation.

‡ Total metabolic tumor volume.
