# Supplementary material for: Baseline 18F-FDG PET/CT Radiomics in Classical Hodgkin’s Lymphoma: The Predictive Role of the Largest and the Hottest Lesions
Source: Diagnostics (Basel). 2023 Apr 11;13(8):1391. doi: 10.3390/diagnostics13081391 (PMC10137254; doi:10.3390/diagnostics13081391)
Supplement: Supplementary file 1 [file diagnostics-13-01391-s001.zip › Triumbari_Supplemental_Table S3.pdf]

SUPPLEMENTAL TABLE S3. Best bivariate radiomic models for 24-months progression free survival prediction (whole cohort)

Covariate 1	Covariate 2	mAUC*	SD† AUC
TMTV‡	Lesion_B.F_rlm.r.perc	0.744	0.1253
Lesion_A.F_cm.2.5Dmerged.corr	Lesion_B.F_szm.szlge	0.730	0.0849
TMTV	Lesion_B.F_ morph.pca.least	0.729	0.0931
TMTV	Lesion_A.F_cm.2.5Dmerged.corr	0.710	0.0715
TMTV	Lesion_B.F_morph.av	0.710	0.0812
Lesion_B.F_rlm.r.perc	Lesion_B.F_cm.energy	0.706	0.0899
TMTV	Lesion_B.F_morph.pca.minor	0.703	0.1058
TMTV	Lesion_B.F_rlm.lre	0.702	0.1086
Lesion_B.F_cm.sum.entr	Lesion_B.F_rlm.rlnu.norm	0.702	0.1011

* Mean area under the ROC curve.

† Standard deviation.

‡ Total metabolic tumor volume.
