# Supplementary material for: Baseline 18F-FDG PET/CT Radiomics in Classical Hodgkin’s Lymphoma: The Predictive Role of the Largest and the Hottest Lesions
Source: Diagnostics (Basel). 2023 Apr 11;13(8):1391. doi: 10.3390/diagnostics13081391 (PMC10137254; doi:10.3390/diagnostics13081391)
Supplement: Supplementary file 1 [file diagnostics-13-01391-s001.zip › Triumbari_Supplemental_Table S4.pdf]

SUPPLEMENTAL TABLE S4. Best bivariate radiomic models for Deauville Score prediction (Scanner_1 images)

Covariate 1	Covariate 2	mAUC*	SD† AUC
Lesion_A.F_cm_merged.sum.entr	Lesion_A.F_cm.auto.corr	0.951	0.0618
TMTV‡	Lesion_A.F_cm.corr	0.945	0.1419
Lesion_A.F_cm.sum.var	Lesion_A.F_cm_2.5D.energy	0.943	0.1495
Lesion_A.F_cm.info.corr.2	Lesion_A.F_cm_2.5D.corr	0.939	0.0977
Lesion_A.F_stat.range	Lesion_A.F_szm.zsnu	0.937	0.2176
Lesion_A.F_szm.zsnu	Lesion_A.F_rlm_2.5D.hgre	0.931	0.1816
TMTV	Lesion_A.F_szm.glnu.norm	0.925	0.0742
Lesion_A.F_szm.zsnu	Lesion_A.F_rlm_2.5D.gl.var	0.914	0.1263
Lesion_A.F_cm.sum.var	Lesion_A.F_cm_merged.clust.tend	0.912	0.0963
TMTV	Lesion_A.F_cm.2.5Dmerged.clust.tend	0.912	0.1133

* Mean area under the ROC curve.

† Standard deviation.

‡ Total metabolic tumor volume.
