# Supplementary material for: Baseline 18F-FDG PET/CT Radiomics in Classical Hodgkin’s Lymphoma: The Predictive Role of the Largest and the Hottest Lesions
Source: Diagnostics (Basel). 2023 Apr 11;13(8):1391. doi: 10.3390/diagnostics13081391 (PMC10137254; doi:10.3390/diagnostics13081391)
Supplement: Supplementary file 1 [file diagnostics-13-01391-s001.zip › Triumbari_Supplemental_Table S5.pdf]

SUPPLEMENTAL TABLE S5. Best bivariate models for 24-months progression free survival prediction (Scanner_2 images)

Covariate 1	Covariate 2	mAUC*	SD† AUC
Lesion_B.F_szm.lzhge	Lesion_A.F_rlm_rlnu.norm	0.873	0.147
Lesion_B.F_szm.glnu	Lesion_B.Dmax	0.873	0.167
Lesion_B.F_szm.lzhge	Lesion_B.F_rlm.glnu	0.861	0.181
Lesion_B.F_stat.energy	Lesion_B.F_rlm.2.5D.r.perc	0.860	0.142
Lesion_A.F_rlm.sre	Lesion_B.F_rlm.2.5D.rl.entr	0.854	0.188
Lesion_B.F_stat.entropy	Lesion_B.F_stat.Nic.entropy	0.852	0.164
Lesion_B.F_stat.entropy	Lesion_B.F_rlm.2.5Dmerged.glnu	0.846	0.144
Lesion_B.F_szm.z.entr	Lesion_B.F_cm_2.5D.info.corr.1	0.845	0.185
Lesion_B.F_stat.energy	Lesion_A.F_szm.2.5D.zs.var	0.844	0.150
Lesion_B.F_cm.info.corr.1	Lesion_B.F_szm_2.5D.glnu.norm	0.844	0.140

* Mean area under the ROC curve.

† Standard deviation.
